# The origin, function and diagnostic potential of extracellular microRNA in human body fluids

**DOI:** 10.3389/fgene.2014.00030

**Published:** 2014-02-12

**Authors:** Andrey Turchinovich, William C. Cho

**Affiliations:** ^1^Molecular Epidemiology Group, German Cancer Research CenterHeidelberg, Germany; ^2^Department of Gynecology and Obstetrics, University Women's Clinic HeidelbergHeidelberg, Germany; ^3^Department of Clinical Oncology, Queen Elizabeth HospitalKowloon, Hong Kong

**Keywords:** body fluids, diagnostic, cell communication, biological fluids

Over the past several years extracellular microRNAs (miRNAs) have been found in all mammalian body fluids such as blood plasma, saliva, urine, milk, seminal plasma, tears, and amniotic fluid as circulating entities capable of predicting the course of a wide range of diseases. Great progress has been made so far in understanding their existence. However, the mechanism of generation and the biological role of extracellular miRNAs remain questionable. Current theories regarding origin and function of extracellular miRNAs suggest that these miRNAs can be either non-specific “by-products” of cellular activity and cell death, or “on-purpose” released cell-cell signaling messengers.

The goal of this Research Topic is to bring together the accumulating knowledge about the extracellular miRNAs function and their utilities for disease diagnostics. We have collected 12 highly selected articles aiming to shed light on the origin and function of circulating miRNAs. We intended to transmit a balanced view on the topic by collecting both research and review articles from the leading scientists in the field of circulating nucleic acids. These papers provide either focused set of experiments or thorough literature analysis with the goal to defend certain concepts. Some researchers have expressed an opinion, strengthened by a number of evidence, that circulating miRNAs can be key mediators of various cell-cell communication processes. Other authors were defending an opinion that extracellular miRNAs can be merely by-products of non-specific cellular activity. While both theories can, in fact, be true, our aim was to secure that the both points of view are expressed.

Several papers touch on the critical methodological concerns associated with the analysis of miRNAs in biological fluids. Dr. Witwer and co-authors described an optimized protocol for isolation of miRNAs from liquid samples (Mcalexander et al., 2013). They showed that some commercial kits are superior to others for the recovery of miRNA from biological fluids. Dr. Cheng and colleagues have presented an excellent review of methods used for deep sequencing of small RNA species in the biological fluids (Cheng et al., 2013). The potential use of miRNA deep sequencing for biomarker discovery and its translation into clinical practice is also discussed. The reports of Dr. Kirschner and colleagues touches on the challenges in analysis, quantification and reporting of the data on the circulating nucleic acids (Kirschner et al., 2013a,b). They have demonstrated a significant impact of hemolysis, which frequently occurs during blood plasma collection, on extracellular circulating miRNA content. Thus, when investigating circulating miRNA in blood plasma and serum it is critical to avoid the hemolysis and to account for all possible artifacts derived from it. Especial branch of our topic is dedicated to the extracellular viral miRNAs. Thus, Dr. Lagana and co-authors provided an excellent state-of-the-art coverage toughing current knowledge and perspectives of the viral circulating miRNAs (Lagana et al., 2013). Virus-encoded miRNAs can be secreted by virus-infected cells and, possibly, be transferred to the recipient cells. The functional consequences of such transfer and the diagnostic potential of viral miRNAs are thoroughly discussed. Finally, Dr. Oliviery and co-authors provided some insights into inflammatory miRNAs in the circulation and their association with age-related diseases (Olivieri et al., 2013). Inflamma-miRs, a relatively small number of miRNAs that are involved in regulation of inflammation, and which mainly target the TLRs/NF-κB pathway, have been recently found in cell-free blood circulation. Interestingly, age-related inflammatory diseases are frequently accompanied by deregulation of most circulating inflamma-miRs in the plasma. Thus, circulating inflamma-miRs could have diagnostic/prognostic relevance in such diseases.

We hope that our Research Topic will raise further attention to this emerging field. Enormous potential of circulating nucleic acids for the prediction of a wide range of diseases is also mentioned. The spectrum of the disorders which can be diagnosed on their early stages using the analysis of circulating miRNA in human bio-fluids biopsies includes: prostate cancer, Alzheimer disease, cardiovascular diseases, viral infections, aging and age-related disorders, etc.

Despite a large number of promising studies, the field of extracellular nucleic acids is still in its infancy. Further advancement of more sensitive and specific methods for detecting and analysis of circulating small RNAs is warranted for addressing the questions raised in this Research Topic. We envision that this Research Topic will attract bigger interest into the field of extracellular miRNAs and encourage the perusal of more in-depth investigations which are translating these molecules into biomarkers for disease diagnostics.
